# The Effect of Grazing Level and Ageing Time on the Physicochemical and Sensory Characteristics of Beef Meat in Organic and Conventional Production

**DOI:** 10.3390/ani11030635

**Published:** 2021-02-27

**Authors:** Isabel Revilla, Javier Plaza, Carlos Palacios

**Affiliations:** 1 Area of Food Technology, E.P.S. of Zamora, University of Salamanca, Avenida Requejo 33, 49022 Zamora, Spain; irevilla@usal.es; 2 Area of Animal Production, Faculty of Environmental and Agrarian Sciences, University of Salamanca, Avenida Filiberto Villalobos 119-129, 37007 Salamanca, Spain; carlospalacios@usal.es

**Keywords:** meat quality, grazing, calves, organic, beef, concentrate

## Abstract

**Simple Summary:**

There is an increasing demand for meat products with high nutritional quality and produced through eco-friendly and sustainable systems, such as organic production. Therefore, in this work, it was proposed to study how the main characteristics of nutritional quality of beef are modified according to the feed management system, in terms of grazing level, and the ageing time of the meat. We found that the increase in pasture intake caused calves’ meat to present lower values of fat oxidation and, simultaneously, higher values of healthy fatty acids for humans. In the case of samples from organic farming, these showed a higher fat content and lower moisture, besides being the darkest samples and those with the lowest score regarding flavour quality in the tasting panel. Furthermore, it was proved that the increase in ageing time resulted in a general improvement of the sensory characteristics of the meat samples, especially those from the animals that had consumed more pasture. Therefore, we suggest extending the ageing period of beef since there is a clear tendency to increase the tenderness and juiciness of beef meat.

**Abstract:**

This study investigated the influence of the production system (conventional vs. organic), the grass consumption level and the ageing period (7 and 14 days) on beef quality. Three groups of samples from Limousin × Avileña calves were analysed: F100, formed by animals fed 100% on forage; F74, formed by animals fed on an average amount of forage of 74%; and F35, formed by animals fed on straw fodder (35%) and concentrate (65%). The results showed that the higher the grass content, the lower the fat oxidation and the higher the n-3 content, but also the higher the SFA level, the initial Warner-Bratzler shear force (WBSF), and the more residue it leaves on chewing. As for the effect of production system, organic samples showed higher intramuscular fat content and lower moisture and MUFA content. These samples were darker and showed lower values for flavour quality. Among the organic samples, F100 had higher n-3 and CLA content and higher values for colour, hardness, odour and flavour quality. Increased ageing time may improve the sensory characteristics of the meat, especially in the case of the F100 samples. The results pointed out that F100 samples aged at least 14 days showed the best physico-chemical, nutritional and sensory characteristics.

## 1. Introduction

In recent years, the intensification of production has raised issues of eco-friendly production in addition to animal health and welfare. Although these are costly goals in terms of both capital and labor [1], an increasing number of consumers are willing to pay higherprices for higher quality products, the production of which is sensitive to both the environment and animal welfare [2]. This could allow farmers to opt for systems in which minimizing production costs is not a priority [3].

Usually, the benefits of organic production systems are mainly related to eco-friendly production and a high standard of animal welfare [4]. However, the production system does not necessarily determine livestock health and product quality but rather the specific management of the farms themselves [5,6]. Therefore, livestock management in organic production is extremely important as many studies claim that there is a close relationship between animal welfare and meat quality [7,8,9].

Meat quality standards are strongly influenced by antemortem factors [10], such as genotype, gender, weather and transport conditions, stress and slaughter methods, but feed is considered the most important of these [11]. They occur from the time the animal leaves the farm and is taken to the slaughterhouse until it is slaughtered. As far as post-mortem factors are concerned, the most important is ageing [12]. The latter is a natural process in which endogenous enzymes (cathepsins and calpains I and II) improve the texture and organoleptic characteristics of meat. It is well known that ageing produces meat tenderization resulting from proteolysis, i.e., the fragmentation of myofibrillary proteins [13]. In addition, the peptides and amino acids generated during proteolysis contribute to the development and enhancement of meat flavour [14]. Ageing is determined by both the animal species and the type of muscle involved [15]. Beef requires longer ageing periods to acquire suitable culinary qualities, ranging from 10 to 21 days [16]. It is consequently necessary to optimize the ageing time in each case, which will depend on the breed [17] and the slaughter age of the animal [18]. Tenderization is therefore more intense in older and heavier animals owing to the greater action of proteases [19].

The animals’ diet is a major factor in overall meat quality [20]. As energy intake increases, therefore, the meat and bone percentages in the carcass decrease and the fat percentages increase. However, diets high in nitrogenous substances increase the growth rate and protein content and in turn decrease the lipid content [21]. Regarding the influence of grazing, some studies point out that meat finished on pasture shows a slightly higher pH, which presumably would increase its water holding capacity [22,23]. Concerning colour, some authors [24] suggest that the variation registered in the chromatic indexes depending on the type of food is not great and that its differences are not significant, whereas others [25,26] have observed that meat from calves fed on grass has a darker colour. In addition, a grass-fed diet affects parameters such as lipid oxidation because it contains a higher amount of vitamin E [27]. Another important effect is that grass-fed finishing improves the fatty acid profile of meat [28,29], which leads to an increase in the unsaturated/saturated ratio, a decrease in the n-6/n-3 ratio, and increased levels of conjugated linoleic acid (CLA) [30].

In this context, the production systems under European organic production legislation are those which most strongly support the establishment of feeding systems in which forage is the main (if not the only) component of the diet. In particular, these European standards require that the forage intake should cover at least 60% of the total intake measured in terms of dry matter [31]. Taking into account the above, this forage intake would lead to a change in the physicochemical, technological, and sensory characteristics of veal, with this change being more pronounced the higher the forage intake present in the diet [32,33].

Nowadays, little further research is being carried out into the comparison between the composition and quality assessment of beef from organic production [34,35] versus that from conventional production systems [31,36]. In the European Union, beef consumers tend to prefer the flavour of beef produced under less intensive production systems [37]. The growing concern for health also encourages this, as studies have shown that organic beef has a lower content of saturated fat, trans fat, and cholesterol but contains a higher proportion of conjugated linoleic acid (CLA) and n-3 fatty acids [38], the latter group being highly beneficial to human health [22].

Considering the above, the initial hypothesis proposed in this study is that the production system (conventional vs. organic), the feeding regime regarding grass consumption level and the ageing period are key factors affecting the physicochemical and sensory properties of beef. Therefore, the aim of this study is to determine how these factors can be optimized to achieve the maximum physicochemical and sensory quality of the meat.

## 2. Materials and Methods

### 2.1. Samples

For this study, 18 calves (3 animals per each of the three feeding systems and from the two different trials) were randomly selected from a larger set of animals. The calves from Limousin × Avileña (native breed) were raised in the “Dehesa de la Serna” farm in the province of Avila, Spain, and they were reared free-range with their mothers during their first six months of life in a dehesa ecosystem. The animals were fed on maternal milk during the first two months and gradually began consuming the available natural pasture until they were weaned. Two trials were carried out: the first one was slaughter in June and the second one in October. After weaning (6 months old), they were randomly segregated into three groups as follows: Group F100 formed by animals fed 100% on fodder from certified Organic Farming pastures; Group F74 formed by animals fed during the first five months on a diet consisting of fodder (80%), 60% of which was fresh grass and the remaining 40% vetch-oats hay, and on a 50%/50% barley and wheat concentrate (20%). For the last two months, they were fed vetch-oat hay (60%) and barley-wheat concentrate (40%). All feeds were certified by Organic Farming. The average amount of fodder eaten over the whole period was 74%; Group F35 includes animals fed on a diet consisting of straw fodder (35%) and specific feed concentrate (31% barley; 9% soya flour; 10% alfalfa hay; 10% wheat bran; 40% wheat flour) for conventional feeding troughs (65%). The nutritional characteristics of the diet of each group are shown in Table 1.

In each trial, 3 calves of each group were randomly selected for slaughter. Then, eighteen animals (6 of each group) were slaughtered when they were 13 months old with a live weight ranging from 250 to 300 kg. Two samples of the *Longissimus thoracis* muscle between T6 and T8 from the left side were taken at 24 h post-mortem from each of the animals and sent to the laboratory of the Area of Food Technology of the EPS of Zamora. The samples were dry-aged in darkness to avoid the pro-oxidant action of light. For each animal, one sample was analysed after 7 days of ageing, and another was kept refrigerated at 4 °C for 14 days of ageing. After the ageing, the samples were vacuum-packaged and kept frozen at −18 °C until the time of analysis.

### 2.2. Analysis of Physicochemical Quality

Before analysis, the meat was thawed under tap water to an internal temperature of 16–18 °C. The pH of the meat was measured at 18 °C with a Crison pHMeter Basic 20 (Crison, Barcelona, Spain) equipped with a penetration electrode. The expressible juice was measured according to a modification of the method of Grau and Hamm [39], as described by Pla [40]. Intramuscular fat (ether-extractable) was determined according to the Soxhlet method, and the moisture content was determined by oven-drying in accordance with the ISO 1443 (1973) norm. The ash content was determined by incineration in a muffle according to ISO R936 method. Fat oxidability was measured to determine the thiobarbituric acid reactive substance (TBARS) content of the samples according to the method of Buege and Aust [41] and expressed as mg of malonaldehyde per kg of sample. All the determinations were carried out in duplicate.

Meat colour was measured with a MiniScan XE Plus Spectrophotometer (Hunter Lab, Reston, VA, USA) after 1 h blooming at 4 °C with a 25 mm measuring head and diffuse/8° optical geometry. CIELab parameters were determined for the CIE illuminant D65 and 10° standard observer conditions. The measured parameters were lightness (L*), redness (a*) and yellowness (b*). The determinations were carried out in triplicate.

The texture was measured after the cooking losses were determined using, for both, the methods described by Revilla and Quintana [17]. A texture analyzer Ta-Xt2i (Stable Micro Systems, Godalming, England) equipped with a Warner–Bratzler device was used. Prismatic samples of dimensions 1 × 1 × 4 cm were prepared using a scalpel and by cutting the piece with the muscle fibres parallel to the longitudinal axis of the sample. In these prismatic samples, the necessary shear force used by the device to cut them (in this case in the transverse direction to the longitudinal axis of the fibres) completely was measured. The crosshead speed was 1 mm/s. The Warner-Bratzler shear force (WBSF) determinations were carried out in triplicate.

### 2.3. Analysis of Fatty Acids and Cholesterol

The fatty acid composition of lipids was determined according to the method described by Lurueña-Martínez [42]. Extracted fatty acids were methylated with KOH 0.2 M in anhydrous methanol and then analysed by gas chromatography (GC 6890 N, Agilent Technologies, Santa Clara, CA, USA) using a 100 m × 0.25 mm × 0.20 µm fused silica capillary column (SP-2560, Supelco, Inc, Bellefonte, PA, USA). One microlitre was injected into the chromatograph, which was equipped with a split/splitless injector and a flame ionization detector (FID). The oven temperature program was started at 150 °C followed by increases of 1.50 °C/min up to 225 °C, at which point it was maintained for 15 min. The temperature of the injector and detector was 250 °C. The carrier gas was helium at 1 mL/min, and the split ratio was 20:1. The different fatty acids were identified by the retention time using a mixture of fatty acid standards (47885-U Supelco, Sigma-Aldrich, Germany) and a mixture of CLA (0563, Sigma-Aldrich, Germany). Fatty acid contents were calculated using chromatogram peak areas and were expressed as g per 100 g total fatty acid methyl esters. 

Cholesterol was extracted by boiling meat samples under reflux in a 50 mL volumetric flask with methanolic potassium hydroxide in the presence of isopropanol. The cholesterol content was determined by enzymatic oxidation (Boehringer Mannheim/R-Biopharm kit, Darmstadt, Germany). All analyses were performed in triplicate.

### 2.4. Sensory Analysis

The QDA (Quantitative Descriptive Analysis) method was used for the sensory characterization, which allows the generation of a multidimensional quantitative model of the attributes to be assessed [43]. Six assessors trained in non-hedonic sequential perception were used. The panelists were between 40 and 55 years old; 50% were women and 50% were men.

In this study, we used some attributes that had been obtained in previous research [17] referring to the effect of the breed and the ageing period on the sensory quality of Aliste veal. These attributes assess the appearance (external colour, internal colour), the texture (toughness, juiciness, fatty sensation, residue after chewing), the odour (odour intensity, odour quality, anomalous odours) and the flavour (flavour intensity, flavour quality, anomalous flavours). The specific training included tasting sessions using photographs (references) for appearance attributes and standardized references for odour, flavour, and texture attributes [44]. Qualification sessions were subsequently held in order to calculate the repeatability and reproducibility of the panellists, as previously described by Pérez-Elortondo [44]. When the panel as a whole presented for all the attributes a standard deviation of <1, the sensory analysis was carried out. A 10-point scale was used, in which 0 referred to the absence of the parameter and 9 to the maximum intensity for each of the attributes.

The tests were carried out in a sensory analysis laboratory with individual cabins isolated from the outside and from the sample preparation room. Samples of the *Longissius dorsi*, approximately 3 cm thick, were used for tasting. First, they were cooked wrapped in aluminium foil on a grill until they reached a centre piece temperature of 70 °C. They were then cut into 2 × 2 × 2 cm pieces and kept at 60 °C until being served within 10 min after cooking. Crackers and distilled water were provided to cleanse the panelists’ palates between samples [45].

### 2.5. Statistical Analysis

The significance of the effects of the rearing system and the ageing time and their interaction (rearing system × ageing time) were obtained by using a Multivariate Analysis of Variance (MANOVA) fitted to a General Linear Model (GLM). Means and standard deviations of the mean were calculated for all variables. The statistical significance of each factor was calculated at the α = 0.05 level using the F-test. The Tukey test was used to test for statistically significant differences between samples regarding the rearing system. All statistical analyses were carried out using the SPSS Package 23 (IBM, Chicago, IL USA).

## 3. Results and Discussion

### 3.1. Physicochemical Composition

The moisture and fat content were significantly affected by diet but not by ageing, as is shown in Table 2. When moisture increases, fat content trends to a decrease and vice versa, while the protein remains constant [46]. Furthermore, these results show that the organic meat samples (F100 and F74) had a significantly higher fat content than the conventional samples (F35). This result is not consistent with those found by other authors [36,47], who found higher fat content in conventional samples than in organic samples.

When the two organic feeds are compared with each other, the higher the grazing level, the higher the moisture content, which was in agreement with the results reported by other experiences [48] for animals fed different levels of grass. On the other hand, the ash content shown in Table 2 was significantly affected by the rearing system, and it was higher for F100 samples. This finding does not coincide with previous results, which found no differences between animals finished on grazing or silage [49] for either organic calves or conventional ones [50]. The interaction was also significant because ash content increased with ageing time for F100 and F35 groups, contrary to the F74 group, which showed the opposite behaviour. This result is related to other compositional changes rather than the increase in minerals [51].

Regarding the technological quality parameters shown in Table 2, a significant effect of the diet on the pH and the expressible juice is observed. However, this effect is not observed in cooking losses. It has been demonstrated that decreases in pH result in a poor water holding capacity by the myofibrillary proteins of the muscle [52], which is usually associated with greater cooking losses [53]. If we compare the two organic groups, it can be seen that the samples of the F74 group had a significantly lower pH value than the samples of the F100 group, which coincides with other scientific results [54]. Moreover, as ruminants are well adapted to eating predominantly fodder-based diets, the increase in grain in their diet leads to an increase in ruminal acidity [55]. Although conventional animals (F35) had a pH lower than those fed grass (F100) but higher than F74 animals, the differences were not statistically significant. The pH values were correlated with the cooking losses in such a way that the lower the pH, the higher the cooking losses, but, although higher losses were also observed in group F74, there were no significant differences between the groups.

On the other hand, it was observed that the expressible juice tended to increase or decrease proportionally with the pH. The expressible juice was therefore significantly lower in organic grain samples (F74) followed by conventional samples (F35), without showing significant differences, and the highest WHC was observed in calves with 100% grass (F100). Although, usually, the higher the pH, the lower the expressible juice [18], other authors did not find a direct correlation between pH and drip losses, which were higher in samples from steers only fed with grass [12]. 

Ageing produced a significant decrease in pH in all cases. It was observed [56] that when the pH was high on day 7 (around 5.7), there was a slight decrease in the pH from day 7 to day 21, which coincide with the results recorded in this case on the 14 days studied. However, this drop in pH due to ageing was not associated with either the water holding capacity or with the cooking losses, as previously reported Grayson et al. 2014 [57]. The data obtained in the analysis of expressible juice ranged from 14 to 21% with an average value of 16.9%, lower than that found in the literature, which indicates values between 20 and 30% of expressible juice [56,58,59].

### 3.2. Instrumental Colour and Texture

Regarding colour parameters shown in Table 2, the L* and a* values were similar to those found in the literature, while the b* value was slightly higher [22,31,60]. The results show that diet did not have a significant effect on any of the colour parameters, although higher a* values were observed for organic samples (F74 and F100) and F100 showed the lower L* value. 

Ageing had a significant influence on both the lightness and the yellow colour, and an increase in both could therefore be observed. This result has been widely reported [61,62] and is due to the formation of metamyoglobin to produce a paler and browner colour [63]. A significant interaction between ageing and rearing system was observed for the a* parameter, due to the decrease in red colour shown by F34 samples during ageing, while the a* values of F100 and F74 were higher at day 14, probably due to the higher myoglobin content of these samples, as observed by Irurueta et al. (2008) [62] in muscles with higher myoglobin content [64].

Table 2 shows the Warner-Bratzler shear force of the samples, which was significantly affected by the rearing system, so the higher the percentage of forage, the lower the WBSF value, which was clearly observed at 7 days of ageing. These results agreed with the majority of studies, indicating that as the grazing level in the animals’ diets increases, so does the toughness [12,22,39]. A further factor to consider is the hardening of the animals’ muscles due to the exercise they do if they have to walk to obtain food. The meat will therefore be tougher [65] as myofibrillary proteins are more important than connective tissue in determining toughness [66].

The data set showed that as ageing progresses, toughness decreases, which is in agreement with several previous studies [17,23,67]. This effect is due to breaks at the junction of the I-band and Z-disk, as well as the degradation of myofibrillar and cytoskeletal proteins caused by the enzymatic action of calpain and calpastine [68]. It was also observed that after 14 days, the organic samples with a higher percentage of forage underwent a significant decrease in the shearing force (*p* < 0.05), while for conventional samples, there were no significant differences in this parameter between both sampling points. The intense softening and the weaker change in colour observed in the F100 samples at this ageing moment suggest the need to expose the meat from animals with greater mobility and higher grazing level to longer ageing periods. Other studies have shown that the optimum time of meat ageing depended on diet [48].

### 3.3. Fat Oxidability

The results found in this study, which are shown in Table 2, are in general lower than those described by other authors [56,69]. Concerning the effect of the diet, it was observed that the higher the proportion of grass, the lower the content of reactive substances to thiobarbituric acid (TBARs), so organic meats showed less lipid oxidation than conventional meats; this difference is statistically significant. This result can be explained by the fact that fresh fodder contains higher concentrations of α-tocopherol than diets with concentrates [70]. For this reason, several authors [71,72] found that the oxidative stability of the muscles of grazing animals was significantly higher than in the meat of animals fed concentrates.

Ageing did not significantly affect TBAR values because the samples were vacuum-packed and in darkness to avoid the pro-oxidant action of light. These results coincide with some scientific results [56] but differ from others [69], in which higher TBAR values were found only in samples of animals fed grain after storage.

### 3.4. Fatty Acid Profile and Cholesterol Content

The results (Table 3) show that the predominant fatty acids were oleic, palmitic and stearic acids (C18:1, C16:0 and C18:0), which is in line with other authors’ results for organic calves [50] or calves under extensive or semi-intensive breeding systems [73,74]. However, when the finishing diet is part of an intensive rearing system or includes high percentages of grains or concentrates, the major fatty acid is stearic followed by oleic and palmitic acids [50]. The levels found for the various fatty acids studied fell within the ranges indicated in the literature [75]. The high contents of C14:0 and C14:1 found in some of the samples analysed are noteworthy. However, in general, linoleic acid (C18:2n6) showed lower contents than those indicated in the literature (9.86–23.7%).

The results showed that there were significant differences in eighteen out of twenty-seven fatty acids analysed, and the fatty acids that showed a significant effect of the rearing system were almost the same as those found in the scientific literature [28,76]. 

The F100 samples presented significantly higher values of C18:3n3 and C20:5n3, while F74 samples showed the highest levels of C22:5 n3 and C20:4 n:6. These results coincide with those found by some authors [28,74,76], who point out that feeding with forage significantly increases C18:3n3 and C20:5n3 but also C20:4n6 acids. The highest level of C22:6 n3 was found for F74 samples; this is in agreement with the results reported by Fruet et al. [28], which do not show a linear increase for any of the n-3 fatty acids with the increase of forage in the diet. In this study, this linear increase was only observed for C20:5 n3, agreeing with Alfaia et al. [76]. Some authors [28] point out that feeding forage increases not only C18:3 n3 in the diet but also the rumen transit time. This fact allows a higher biohydrogenation of this fatty acid [77] resulting in similar levels of C18:3 n3 acid for F35 and F74 samples. Indeed, this fact is responsible for the increased deposition of C18:2n6 in F74 samples, which show similar levels of this fatty acid as F35 samples. The higher levels of these fatty acid found in the grain fed diets samples is in agreement with previous works because C18:2n6 is one of the main fatty acids in cereal grains [78], although no significant differences were observed among diets as reported by Alfaia et al. [76].

The higher concentrations of C18:3n3 in fresh forage (F100 diet) is also responsible for higher but not significant levels of C18:2 9c11t found in these samples. Some authors suggest that different concentrate sources such as soybeans can increase the deposition in the lean of C18:2 9c11t, pointing out the importance of the supplementation on the concentration of this compound [79]. There were no statistically significant differences between organic and conventional samples, which coincides with the results found in other studies [50,58], in which it was also found that there were no differences between the different finishing systems.

The conventional meat samples that include the higher percentage of concentrates showed significantly higher contents of C18:1 n9c and its isomer C18:1 n9t. This result is in agreement with those reported by Fruet et al. [28], Horcada et al. [80] and Alfaia et al. [76], and it is the result of the higher oleic contents in the grains but also due to the increase in the delta9 desaturase activity in ruminants fed high-grain diets [81] and probably qualified by higher production of insulin [82].

Regarding saturated fatty acids, the F74 samples presented significantly higher values of the short-chain saturated fatty acids (C6:0, C8:0, C10:0 and C12:0) while F100 samples showed the highest values of C14:0, C16:0 and C18:0. Other works [29,34,78] found that meat from animals fed in pasture showed significantly higher values of C18:0, which was possibly associated with a rapid hydrogenation of C18:3n3 in the rumen. The concentrations of the saturated fatty acids were higher than expected. This could be due to the fact that the higher the intramuscular fat content, the higher the proportion of triacylglycerols and the lower the phospholipids, and as a result, lower percentages of PUFA and higher proportions of MUFA and SFA than expected for F100 and F74 samples were found [76].

As regards the results obtained for the fatty acids grouped by their degree of unsaturation, it was noted that the content of the forage led to significant differences in the total content of saturated (SFA) and monounsaturated (MUFA) fatty acids and in the total content of n3. The organic samples therefore presented significantly lower levels of MUFA and significantly higher levels of SFAm whereas the samples from conventional animals contained a higher percentage of oleic acid. The higher proportion of MUFA observed for the F35 group is due to the higher C18:1 content, as mentioned above. The F74 group showed a higher level of PUFA, although it was no significant. According to certain scientific results [83], the explanation for this higher PUFA percentage in meat from pasture-fed animals may be the higher protection of fatty acids in fresh grass from ruminal biohydrogenation relative to that of grain or silage. Moreover, this increase in the meat PUFA percentage could also be a result of the presence of secondary plant metabolites on spontaneous pastures, which might inhibit microbial biohydrogenation activity within the rumen [84]. Significantly higher contents of n3 were observed for F100. These differences are a consequence of the fatty acid composition of the diet, with α-linolenic acid (C18:3, the n-3 series precursor) being the major fatty acid in grass lipids. Indeed, as noted previously [85], despite the hydrogenating effects of rumen microorganisms, diet can increase the content of n3 muscle fatty acids in beef, particularly of C18:3.

Owing to the increase in n-3 fatty acids, the lower the n-6/n-3 ratio, the higher the percentage of grass in the diet. Other studies have reported that higher levels of concentrate in the diet increase the n-6/n-3 ratio of meat [83,86] and that the ratio decreased as the proportion of pasture increased [76]. The n-6/n-3 ratio is widely used to assess the nutritional value of fat for human consumption. According to the World Health Organization, this ratio should be below 4:1. Taking into account the results, the F100 group was below the recommended level which was similar to that previously reported for animals feeding on pasture [71,73,78].

Finally, the cholesterol content showed no statistically significant differences between the three groups, although the F100 samples seem to trend towards lower levels (*p* < 0.1). This result is in agreement with that previously reported by other authors [87,88], who failed to find any significant differences owing to the feeding strategy. However, some others [89] reported slightly lower cholesterol levels for pasture-finished steers as observed for F100 in this study.

### 3.5. Sensory Analysis

The results obtained from the sensory analysis were grouped into colour, odour, flavour and texture attributes (Table 4). As regards the colour, both the external and the internal colour intensity was higher in organic than in conventional samples. These results are in agreement with those observed for the instrumental colour and coincided with the previous studies indicating that grain or concentrated finished meats are lighter than those finished with grass [22,48]. This has been linked firstly to the higher myoglobin content of grazing animals owing to their greater physical activity [90]. 

As far as the effect of ageing is concerned, it can be noted that as the ageing progresses, the external colour is hardly affected; indeed, there are no statistically significant differences. Although there are no significant differences in the internal colour either, it could be expected that the organic samples move towards lightening (*p* < 0.1) as the ageing process advances. This result coincides with that observed in the evolution of the L* parameter and is related to the increase in lipid oxidation and the probable increase in myoglobin oxidation [70], which is greater the lower the pH [64].

The results concerning odour intensity do not show any statistically significant differences in agreement with what was found by other authors [14,23,91]. It should be emphasized that after 7 days, the F100 samples had a lower odour intensity than the samples with grain in the diet. The conventional F35 samples, both overall and more particularly after 7 days of ageing, were those with the highest odour quality. These results coincide with those found by other authors [92], who state that the flavour of samples from high-grass-content diets is related to the deposit of alkylphenols in sheep fat (methylphenols, isopropylphenols and other phenolic compounds). In other studies [93], the volatile compounds of bull meat after cooking were assessed, and it was concluded that animals fed with grass had a “green” aroma, which is related to hexanal derived from the linoleic fatty acid, and higher contents of (E,Z)-2,6-nonadienal with a “cucumber-like” odour derived from linolenic acid. These results are confirmed by the lower initial score of abnormal aromas in conventional samples with less grass.

However, ageing affects organic and conventional samples in different ways, generating in the former an increase in the scores of odour intensity and a decrease in the latter, which justifies the absence of significant differences due to ageing. This increase in organic samples coincides with that found in other studies [14,23] and is due to the fact that during ageing, there is a more intense development of the aromas produced by enzymatic (proteolysis or lipolysis) or non-enzymatic biological reactions such as fat oxidation. Regarding odour quality, a significant decrease in this attribute was observed, mainly due to the changes observed for F100 and F35 samples, which had higher initial values for this parameter. This change in odour quality is related to the increase in the score of abnormal odours, although this was not statistically significant.

Regarding toughness, the differences attributed to the different diets were not statistically significant. This result is due to the different effect of ageing according to the type of sample. The toughness of the F100 and F35 samples therefore decreased, in contrast to the F74 samples, in which this parameter increased although the differences were not statistically significant. 

As for juiciness, F35 samples had a lower initial value than the F100 and F74 samples. This does not coincide with other results found in the scientific literature [14,23], in which organic samples started from lower juiciness values than conventional ones, and it may be related to the lower intramuscular fat of F35 samples [94]. However, during ageing, F34 samples experienced a strong increase in juiciness, unlike the organic samples, which increased either slightly (F100) or not at all (F74). This result could be attributed to differences in enzymatic degradation of myofibrillar and cytoskeletal proteins that may differ depending on the physical activity of the animals [95,96].

This made the diet × ageing interaction significant (*p* = 0.002) but not each of the factors separately. Juiciness and toughness are closely related, such that the lesser the toughness, the more and the faster the juices are released when chewing [97], as is shown in this study. 

As regards the fatty sensation, very low scores were obtained in all cases, which correlates with the low-fat content of all the samples analysed. For this parameter, there were no statistically significant differences either due to the production system or to the diet or the combined effect of both. Finally, the F100 samples were those leaving more residues after chewing, followed by the F74 samples and finally the F35. The ageing time did not show a significant effect; it is, however, noteworthy that this parameter decreased by 14 days in the F100 and F35 samples, coinciding with the decrease in toughness.

Within the attributes of flavour assessment, the flavour intensity was clearly affected by feeding and presented a higher value in diets including grain intake (F74 and F35); these differences were statistically significant (*p* < 0.05). These results disagree with previous findings [14,23], which suggest that samples of animals fed with a higher proportion of grasses present a more intense flavour. However, in other studies, it was shown that the flavour in organic samples presented lower values than those found in conventional samples [98,99], as occurs in this case. Although flavour intensity tended to increase, the changes generated by the ageing process were not statistically significant.

As far as flavour quality is concerned, the F35 conventional samples obtained higher scores than the F100 and F74 organic samples, and these differences were statistically significant. The differences are particularly noticeable at 7 days as in the case of odour quality, since there is a close correlation between odour and flavour. As mentioned above, the increase in components deriving from unsaturated fatty acids generated by the consumption of grass causes the appearance of “green” [93], “grass”, “animal”, “faecal”, “sheep”, “stable” and “milky” odours and flavours [100]. In addition, the presence of grass-based compounds such as methylphenols, isopropylphenols and other phenolic compounds provides a certain “animal odour” and consequently generates the perception of unpleasant flavours for the consumer [101]. The ageing process caused the flavour quality to decrease in all types of samples analysed, but these differences were not statistically significant. Finally, the presence of abnormal flavours is directly correlated with flavour quality, as it was the F74 samples that showed the highest values in this parameter; they coincided with the results obtained from measuring flavour quality, in which this group of samples obtained the lowest scores. The differences generated by the rearing system were statistically significant. The increase in abnormal flavours due to the ageing is responsible for the decrease observed in flavour quality, and differing from abnormal odours, the effect of ageing was statistically significant.

## 4. Conclusions

The results showed an increasing trend in the initial values (7 days of ageing) of WBSF, the residue after chewing, the total amount of PUFA n-3 and the total amount of SFA, as the percentage of forage increased. Conversely, as the forage content decreased, a decreasing trend was observed in the values of TBARs, the initial odour intensity, and the initial flavour quality. On the other hand, a significant effect of organic vs. conventional production was observed for fat content, moisture and total MUFA, with organic production samples showing a higher intramuscular fat content and lower moisture and MUFA values. Moreover, as the sensory panel evaluation shows, organic samples had higher values for internal and external colour and lower values for odour and flavour quality. 

The greatest differences were found between the organic F100 vs. the F74 finishing systems—the higher the percentage of grass, the higher the ash content, the pH, the moisture, the n-3 content, the CLA levels, the flavour intensity and the odour quality, but this meat was also tougher and darker than F74. This suggests that the quality of organic meat is improved by including higher percentages of grass in the diet. Moreover, the results point to the fact that it could be advisable to increase the days of ageing, especially for 100% pasture animals, since this group showed a significant decrease in WBSF without significant changes in colour, odour and flavour intensity and with no significant changes in flavour and odour quality.

## Figures and Tables

**Table 1:** Nutritional composition of the intakes of the different rearing systems (%Dry Matter).

Group	Raw Matter	Period	%	%CP	%EE	%ADF	%NDF	%Sugar	%Starch	NEg Mcal/KgIDM	%C14:0	%C16:0	%C18:0	%C18:1	%C18:2	%C18:3
F100	Natural pasture	Complete	100	9.80	2.10	40.27	58.01	6.62	2.00	0.57	0.03	0.43	0.06	0.20	0.56	0.54
F74	Natural pasture	Growing	50	4.92	1.07	20.13	29.01	3.30	1.02	0.28						
	Pasture hay	Finishing	24	2.36	0.51	9.84	14.16	1.58	0.49	0.14						
	Barley + Oats	Finishing	26	3.64	0.51	1.99	4.50	0.50	11.03	0.31						
	Total		100	10.92	2.09	31.96	47.67	5.38	12.54	0.73	0.02	0.40	0.05	0.26	0.60	0.42
F35	Cereal straw	Complete	35	1.61	0.32	16.00	24.89	0.00	0.56	0.16						
	Composite feed	Complete	65	10.36	1.68	7.13	18.29	3.50	18.06	0.75						
	Total		100	11.97	2.00	23.13	43.18	3.50	18.62	0.91	0.00	0.23	0.01	0.17	0.65	0.08

CP: crude protein; EE: ether extract; ADF: acid detergent fibre; NDF: neutral detergent fibre, NEg: net energy of gain; IDM: ingested dry matter.

**Table 2:** Physicochemical parameters depending on the rearing system and ageing time (mean ± SD).

Parameter		F100			F74			F35				Significance		
	7	14	Total F100	7	14	Total F74	7	14	Total F35	Total 7	Total 14	R	A	R*A
Ash (%)	1.20 ± 0.11	1.80 ± 0.90	1.50 ^a^ ± 0.69	1.26 ± 0.17	1.10 ± 0.13	1.17 ^b^ ± 0.17	1.15 ± 0.04	1.20 ± 0.05	1.18 ^b^ ± 0.05	1.21 ± 0.12	1.35 ± 0.58	0.011	-	0.006
IMF (%)	5.27 ± 4.82	3.67 ± 2.62	4.47 ^a^ ± 3.86	5.19 ± 5.90	3.70 ± 2.69	4.38 ^a^ ± 4.39	0.93 ± 0.44	1.59 ± 1.18	1.26 ^b^ ± 0.93	3.80 ± 4.72	3.03 ± 2.43	0.006	-	-
Moisture (%)	69.80 ± 1.43	71.80 ± 2.37	70.80 ^a^ ± 2.16	67.37 ± 4.97	65.54 ± 6.65	66.37 ^b^ ± 5.88	72.53 ± 1.96	71.79 ± 1.80	72.16 ^a^ ± 1.87	69.90 ± 3.75	69.45 ± 5.26	0.000	-	-
pH	5.81 ± 0.24	5.58 ± 0.27	5.69 ^a^ ± 0.28	5.58 ± 0.08	5.30 ± 0.21	5.44 ^b^ ± 0.21	5.60 ± 0.28	5.56 ± 0.31	5.58 ^ab^ ± 0.29	5.66 ± 0.24	5.49 ± 0.29	0.001	0.001	-
Expressible juice (%)	16.86 ± 5.98	20.96 ± 6.28	18.91 ^a^ ± 6.38	17.74 ± 3.65	14.13 ± 4.07	15.87 ^b^ ± 4.23	15.94 ± 3.08	16.28 ± 2.21	16.11 ^b^ ± 2.65	16.77 ± 4.33	17.07 ± 5.13	0.015	-	0.005
Cooking losses (%)	17.46 ± 3.48	14.31 ± 3.03	15.89 ± 3.49	20.28 ± 4.09	16.03 ± 3.72	18.15 ± 4.33	17.37 ± 4.09	18.07 ± 2.86	17.72 ± 3.35	18.49 ± 3.91	16.13 ± 3.40	-	0.094	-
L*	37.42 ± 3.53	38.69 ± 4.49	38.05 ± 4.02	38.98 ± 3.24	41.04 ± 3.46	40.05 ± 3.46	37.15 ± 9.39	40.25 ± 3.01	38.70 ± 7.05	37.78 ± 6.32	40.01 ± 3.71	-	0.049	-
a*	14.99 ± 1.52	15.38 ± 2.09	15.18 ± 1.81	14.25 ± 1.23	16.55 ± 2.19	15.44 ± 2.11	15.24 ± 3.61	13.37 ± 2.65	14.30 ± 3.26	14.86 ± 2.47	14.99 ± 2.66	-	-	0.004
b*	13.15 ± 1.34	14.75 ± 2.82	13.95 ± 2.32	13.03 ± 0.83	15.99 ± 1.65	14.56 ± 1.99	13.48 ± 2.42	12.88 ± 2.48	13.36 ± 2.47	13.38 ± 1.75	14.44 ± 2.67	-	0.007	0.001
WBSF (N)	77.19 ± 45.71	37.52 ± 16.83	57.36 ^a^ ± 39.46	42.81 ± 16.86	37.64 ± 17.57	40.23 ^b^ ± 17.10	26.64 ± 3.10	28.47 ± 6.50	27.56 ^b^ ± 5.03	54.36 ± 37.90	35.58 ± 15.61	0.000	0.012	0.005
mg MDA/kg	0.008 ± 0.006	0.011 ± 0.004	0.009 ^b^ ± 0.005	0.020 ± 0.007	0.016 ± 0.006	0.018 ^b^ ± 0.007	0.096 ± 0.066	0.094 ± 0.072	0.095 ^a^ ± 0.067	0.041 ± 0.054	0.039 ± 0.054	0.000	-	-

^a. b.^ Different letters mean statistically significant differences *p* < 0.05, according to Tukey’s HSD test. “-”: *p*-value > 0.05, no statistically significant differences, SD: standard deviation, R: rearing system, A: ageing, IMF: intramuscular fat, MDA: malondialdehyde, WBSF: Warner-Bratzler shear force.

**Table 3:** Fatty acids (as g/100 g of fatty acids) and cholesterol of beef samples (mean ± SD).

Fat Acid	F100	F74	F35	Significance
C6:0	0.681 ^b^ ± 0.857	2.021 ^a^ ± 0.047	0.958 ^b^ ± 0.388	0.005
C8:0	0.992 ^b^ ± 1.288	2.795 ^a^ ± 0.054	1.353 ^b^ ± 0.560	0.011
C10:0	1.099 ^b^ ± 1.357	2.998 ^a^ ± 0.048	1.478 ^b^ ± 0.652	0.013
C11:0	0.154 ± 0.046	0.206 ± 0.001	0.251 ± 0.132	-
C12:0	0.969 ^b^ ± 1.236	2.714 ^a^ ± 0.042	1.380 ^b^ ± 0.356	0.016
C14:0	3.841 ^a^ ± 0.787	2.436 ^b^ ± 0.002	3.124 ^ab^ ± 0.555	0.009
C14:1	1.349 ± 0.381	2.720 ± 0.005	1.221 ± 0.735	0.002
C15:0	0.728 ^b^ ± 0.592	0.406 ^a^ ± 0.000	0.746 ^b^ ± 0.208	-
C16:0	25.376 ^a^ ± 1.191	22.335 ^b^ ± 0.020	22.645 ^ab^ ± 2.371	0.032
C16:1	4.131 ± 0.931	3.613 ± 0.002	3.656 ± 0.394	-
C16:1*t*	0.555 ^b^ ± 0.581	1.615 ^a^ ± 0.026	0.440 ^b^ ± 0.435	0.002
C17:0	1.354 ^a^ ± 0.410	0.866 ^b^ ± 0.002	1.404 ^a^ ± 0.146	0.008
C17:1	0.510 ^b^ ± 0.185	0.650 ^b^ ± 0.003	1.075 ^a^ ± 0.125	0.000
C18:0	16.633 ^a^ ± 5.239	12.433 ^ab^ ± 0.296	10.984 ^b^ ± 1.788	0.020
C18:1 n9t	2.289 ^b^ ± 1.256	1.275 ^b^ ± 0.009	4.600 ^a^ ± 0.915	0.000
C18:1 n9c	31.206 ^b^ ± 2.454	30.574 ^b^ ± 0.218	35.156 ^a^ ± 4.138	0.050
C18:1 n11t	1.075 ± 0.290	1.064 ± 0.014	1.393 ± 0.272	-
C18:2 n6c	2.973 ± 0.555	4.428 ± 0.015	4.249 ± 1.279	-
C18:3 n3	1.058 ^a^ ± 0.428	0.218 ^b^ ± 0.007	0.398 ^b^ ± 0.182	0.000
CLA (C18:2 9c11t)	0.478 ± 0.173	0.305 ± 0.003	0.375 ± 0.099	-
C20:3 n9	0.176 ^b^ ± 0.113	0.400 ^a^ ± 0.000	0.242 ^b^ ± 0.105	0.011
C20:4 n6	0.678 ^b^ ± 0.548	1.465 ^a^ ± 0.003	0.878 ^ab^ ± 0.418	0.036
C23:0	0.088 ± 0.042	0.139 ± 0.008	0.144 ± 0.082	-
C22:2	0.167 ^b^ ± 0.182	0.419 ^a^ ± 0.011	0.213 ^b^ ± 0.110	0.021
C20:5 n3	0.191 ^a^ ± 0.126	0.121 ^ab^ ± 0.001	0.067 ^b^ ± 0.024	0.025
C22:5 n3	0.047 ^c^ ± 0.028	0.211 ^a^ ± 0.000	0.145 ^b^ ± 0.043	0.000
C22:6 n3	0.099 ± 0.099	0.232 ± 0.003	0.132 ± 0.076	-
SFA	52.114 ^a^ ± 3.515	49.465 ^a^ ± 0.499	44.557 ^b^ ± 2.211	0.000
MUFA	41.118 ^b^ ± 2.295	41.515 ^b^ ± 0.182	47.544 ^a^ ± 3.818	0.003
PUFA	6.789 ± 1.549	9.600 ± 0.012	7.897 ± 2.129	-
n-3 PUFA	1.454 ^a^ ± 0.239	0.784 ^b^ ± 0.009	0.744 ^b^ ± 0.237	0.000
n-6 PUFA	4.046 ± 1.034	6.313 ± 0.030	5.341 ± 1.761	-
n6/n3	2.922 ^b^ ± 1.188	8.046 ^a^ ± 0.134	7.166 ^a^ ± 0.725	0.000
Cholesterol	36.262 ± 0.251	39.043 ± 9.963	39.01 ± 10.934	-

^a. b.^ Different letters mean statistically significant differences *p* < 0.05, according to Tukey’s HSD test. “-”: *p*-value > 0.05, statistically no significant differences, SD: standard deviation.

**Table 4:** Sensory attributes depending on the rearing system and ageing time (mean ± SD).

Parameter		F100			F74			F35				Significance		
	7	14	Total F100	7	14	Total F74	7	14	Total F35	Total 7	Total 14	R	A	R*A
External colour	5.07 ± 1.28	5.06 ± 1.54	5.07 ^ab^ ± 1.40	5.65 ± 0.76	5.73 ± 0.87	5.69 ^a^ ± 0.81	4.73 ± 1.32	4.83 ± 1.72	4.78 ^b^ ± 1.50	5.15 ± 1.18	5.26 ± 1.41	0.006	-	-
Internal colour	4.57 ± 1.63	4.43 ± 1.95	4.50 ^ab^ ± 1.77	5.52 ± 1.11	4.97 ± 1.69	5.23 ^a^ ± 1.46	3.54 ± 1.64	4.02 ± 1.85	3.76 ^b^ ± 1.73	4.52 ± 1.68	4.62 ± 1.83	0.001	-	-
Odour intensity	5.38 ± 1.19	5.36 ± 0.93	5.37 ± 1.04	5.47 ± 0.83	6.17 ± 1.07	5.84 ± 1.02	5.85 ± 1.27	5.72 ± 1.01	5.79 ± 1.15	5.60 ± 1.10	5.81 ± 1.05	-	-	-
Odour quality	5.46 ± 1.26	5.00 ± 2.00	5.21 ^ab^ ± 1.68	4.92 ± 1.71	4.93 ± 1.20	4.93 ^b^ ± 1.44	6.28 ± 1.00	5.47 ± 1.09	5.91 ^a^ ± 1.11	5.58 ± 1.47	5.12 ± 1.42	0.010	-	-
Abnormal odours	0.38 ± 0.76	1.00 ± 2.10	0.71 ± 1.62	0.82 ± 1.53	1.00 ± 1.34	0.91 ± 1.42	0.14 ± 0.47	0.44 ± 0.78	0.28 ± 0.64	0.45 ± 1.07	0.82 ± 1.45	-	-	-
Toughness	3.88 ± 1.68	3.70 ± 2.22	3.78 ± 1.95	3.35 ± 1.25	4.02 ± 1.86	3.70 ± 1.62	4.23 ± 1.30	3.58 ± 1.19	3.93 ± 1.27	3.82 ± 1.41	3.79 ± 1.76	-	-	-
Juiciness	3.84 ± 1.40	4.13 ± 2.34	4.00 ± 1.93	4.75 ± 1.42	3.95 ± 1.26	4.32 ± 1.38	3.45 ± 1.90	5.27 ± 1.12	4.29 ± 1.82	4.02 ± 1.70	4.42 ± 1.66	-	-	0.002
Fatty sensation	0.30 ± 0.48	0.40 ± 0.50	0.35 ± 0.48	0.75 ± 0.92	0.47 ± 0.83	0.60 ± 0.87	0.57 ± 0.74	0.55 ± 0.51	0.56 ± 0.64	0.57 ± 0.77	0.48 ± 0.65	-	-	-
Residues after chewing	2.92 ± 1.70	2.86 ± 2.35	2.89 ± 2.04	2.25 ± 0.98	3.13 ± 1.28	2.72 ± 1.22	2.73 ± 1.57	2.66 ± 1.32	2.70 ± 1.44	2.60 ± 1.42	2.91 ± 1.63	-	-	-
Flavour intensity	4.42 ± 0.95	4.43 ± 1.09	4.42 ^b^ ± 1.01	5.50 ± 1.00	5.89 ± 1.18	5.70 ^a^ ± 1.10	5.02 ± 1.53	5.52 ± 0.84	5.25 ^a^ ± 1.27	5.05 ± 1.27	5.38 ± 1.20	0.000	-	-
Flavour quality	4.84 ± 1.51	4.76 ± 1.11	4.80 ^b^ ± 1.29	5.05 ± 1.53	3.82 ± 1.49	4.39 ^b^ ± 1.62	5.71 ± 1.14	5.63 ± 1.28	5.67 ^a^ ± 1.19	5.25 ± 1.41	4.66 ± 1.65	0.000	-	-
Abnormal flavours	0.23 ± 0.59	0.20 ± 0.41	0.21 ^b^ ± 0.49	0.82 ± 1.59	2.86 ± 2.60	1.91 ^a^ ± 2.40	0.23 ± 0.43	0.25 ± 0.54	0.24 ^b^ ± 0.48	0.45 ± 1.07	1.31 ± 2.13	0.000	0.017	0.002

^a. b.^ Different letters mean statistically significant differences *p* < 0.05, according to Tukey’s HSD test. “-”: *p*-value > 0.05, statistically no significant differences, SD: standard deviation, R: rearing system, A: ageing.

## Data Availability

The data presented in this study are available on request from the corresponding author.
